# NLRP1 inflammasome promotes senescence and senescence-associated secretory phenotype

**DOI:** 10.1007/s00011-024-01892-7

**Published:** 2024-06-21

**Authors:** Inés Muela-Zarzuela, Juan Miguel Suarez-Rivero, Andrea Gallardo-Orihuela, Chun Wang, Kumi Izawa, Marta de Gregorio-Procopio, Isabelle Couillin, Bernhard Ryffel, Jiro Kitaura, Alberto Sanz, Thomas von Zglinicki, Gabriel Mbalaviele, Mario D. Cordero

**Affiliations:** 1 Department of Molecular Biology and Biochemical Engineering, Universidad Pablo de Olavide, 41013 Seville, Spain; 2 Instituto de Investigación E Innovación Biomédica de Cádiz (INiBICA), Hospital Universitario Puerta del Mar, Cádiz, Spain; 3 Division of Bone and Mineral Diseases, Washington University School of Medicine, St. Louis, MO 63110 USA; 4 Atopy (Allergy) Research Center, Juntendo University Graduate School of Medicine, Tokyo, Japan; 5 Laboratory of Experimental and Molecular Immunology and Neurogenetics (INEM), UMR 7355, CNRS, University of Orleans, Orléans, France; 6 IDM, University of Cape Town, Cape Town, South Africa; 7 School of Molecular Biosciences, College of Medical, Veterinary and Life Sciences, University of Glasgow, Glasgow, G12 8QQ UK; 8 Ageing Research Laboratories, Newcastle University, Biosciences Institute, Newcastle, UK; 9 CIBER de Enfermedades Respiratorias (CIBERES), Instituto de Salud Carlos III, 28220 Madrid, Spain

**Keywords:** NLRP1 inflammasome, Senescence, SASP, Aging

## Abstract

**Background:**

Senescence is a cellular aging-related process triggered by different stresses and characterized by the secretion of various inflammatory factors referred to as senescence-associated secretory phenotype (SASP), some of which are produced by the NLRP3 inflammasome. Here, we present evidence that the NLRP1 inflammasome is a DNA damage sensor and a key mediator of senescence.

**Methods:**

Senescence was induced in fibroblasts in vitro and in mice. Cellular senescence was assessed by Western blot analysis of several proteins, including p16, p21, p53, and SASP factors, released in the culture media or serum. Inflammasome components, including NLRP1, NLRP3 and GSDMD were knocked out or silenced using siRNAs.

**Results:**

In vitro and in vivo results suggest that the NLRP1 inflammasome promotes senescence by regulating the expression of p16, p21, p53, and SASP factors in a Gasdermin D (GSDMD)-dependent manner. Mechanistically, the NLRP1 inflammasome is activated in response to genomic damage detected by the cytosolic DNA sensor cGMP-AMP (cGAMP) synthase (cGAS).

**Conclusion:**

Our findings show that NLRP1 is a cGAS-dependent DNA damage sensor during senescence and a mediator of SASP release through GSDMD. This study advances the knowledge on the biology of the NLRP1 inflammasome and highlights this pathway as a potential pharmcological target to modulate senescence.

**Supplementary Information:**

The online version contains supplementary material available at 10.1007/s00011-024-01892-7.

## Introduction

Aging generates specific changes associated with cellular senescence, which has been associated to three possible origins: replicative senescence, non-replicative senescence, or stress-induced senescence which, can be prompted by different events such as oxidative stress, oncogenic signaling, or exposure to ionizing radiation. These events induce DNA damage, thereby activating signaling pathways such as the p53 and p16 pathways, which promote cell cycle arrest and initiate senescence-associated programs [1]. This permanent state of cell cycle arrest promotes tissue remodelling during development but leads to the declined tissue regenerative potential and function after injury, and activates inflammation and tumorigenesis in aged organisms [1, 2]. Senescence is associated with a number of distinctive phenotypes, including the development of a pro‐inflammatory response and promotes the production of cytokines, chemokines, proteases, and growth factors, commonly known as the senescence‐associated secretory phenotype (SASP). The SASP is thought to communicate with the immune system and facilitate immune surveillance, stimulating the clearance of senescent or pre‐malignant cells but chronic exposure to the SASP can lead to age‐associated tissue dysfunction [1]. Recent studies demonstrate that chromatin is instrumental in regulating SASP and that stressed cells release extra-nuclear DNA, activating the cyclic GMP-AMP synthase (cGAS)-stimulator of interferon genes (STING) pathway, thereby promoting inflammation [3].

Inflammasomes are intracellular protein complexes involved in almost all human aging-associated complications such as cancer, cardiovascular, metabolic, and neurodegenerative diseases through the production of interleukin-1β (IL-1β) and IL-18 and induction of pryroptosis [4]. These protein platforms comprise sensing proteins (e.g., NOD-like receptor (NLR) family, absent in melanoma-like (ALR) family), the adaptor protein apoptosis-associated speck-like protein containing a CARD (ASC), and procaspase-1. Upon sensing of pathogen-associated molecular patterns (PAMPS) or damage-associated molecular patterns (DAMPS) [4], some NLRs such as absent in melanoma 2 (AIM2), NLRP3, and NLRP1 associate with ASC and recruit pro-caspase-1 and activate it [4, 5]. Active caspase-1 cleaves pro-IL-1β, pro-IL-18, and GSDMD [4]. GSDMD N-terminal fragments form plasma membrane pores through IL-1β and IL-18 are secreted [4]. Excessive pore formation causes pyroptosis, releasing pro-inflammatory intracellular factors such as IL-1α alongside IL-1β and IL-18 [5]. Despite scientific advances in the biology of the NLRP1 and NLRP3 inflammasomes, the role that these proteins play in senescence remain controversial [6–9].

Irradiation of cells or tissues is a model of stress-induced senescence, which we used to determine the role of NLRP1 and NLRP3 inflammasomes in this process [6]. We find that irradiation induces the expression of NLRP1, NLRP3, and SASP. We report that inhibition of the NLRP1 inflammasome, but not the NLRP3 inflammasome attenuates the expression of senescence markers, responses that are cGAS- and GSDMD-dependent.

## Material and methods

### Reagents

Monoclonal antibodies specific for NLRP1 (NBP1-54899), NLRP3 (NBP2-12446) and p21/CIP1/CDKN1A (NBP2-29463) were purchased from Novus Biologicals (Colorado, USA). Similarly, anti-Caspase 1, IL-1β (D3U3E), cGAS (#79978), p16^INK4A^ (mouse: ab189034 and human: 18769S) were obtained from Cell Signaling Technology (Beverly, MA, USA). Finally, IL-6 (sc-32296), p53 (sc-126) antibodies and DAPI were obtained from Santa Cruz Biotechnology (Santa Cruz, CA, USA). Goat Anti-Rabbit IgG H&L (HRP), goat Anti-Mouse IgG, H&L Chain Specific Peroxidase Conjugate, Poly(deoxyadenylic-thymidylic) acid sodium salt (Poly dA-dT), BSA and Triton X-100 were obtained from Merck (Darmstadt, Germany). Val-boroPro—Calbiochem 5314650001 was obtained from Merck (Darmstadt, Germany). Necrosulfonamide was obtained from Sigma-Aldrich (Saint Louis, USA). A cocktail of protease inhibitors (Complete™ Protease Inhibitor Cocktail) was purchased from Boehringer Mannheim (Indianapolis, IN). The Immun Star HRP substrate kit was obtained from Bio-Rad Laboratories Inc. (Hercules, CA). Secondary Alexa Fluor 488 Goat Anti-Rabbit Antibody was obtained from Thermo Fisher (MA, USA). Finally, siRNAs of control and cGAS (AM16708 129125), NLRP3 (4392420 S41555) and NLRP1 (4392420 S22520) were obtained from Invitrogen (Eugene, OR, USA).

### Ethical statements

Animal studies were performed in accordance with the European Union guidelines (2010/63/EU) and the corresponding Spanish regulations for the use of laboratory animals in chronic experiments (RD 53/2013 on the care of experimental animals). All experiments were approved by the local institutional animal care committee.

### Animals

For all experiments, only male mice were used. WT C57/BL6/J, *Nlrp1a*^−/−^*Nlrp1b*^−/−^*Nlrp1c*^−/−^ (*Nlrp1*^−/−^) (C57BL/6J background, provided and originally generated and characterized in the laboratory of Seth L Masters, reference [10]) and *Nlrp3*^−/−^ transgenic mice (C57BL/6J background, provided by Bernhard Ryffel and originally generated and characterized in the laboratory of J. Tschopp, reference [11]), weighing 25–30 g, were maintained on a regular 12 h light/dark cycle. All groups had ad libitum access to their prescribed diet and water throughout the study. Body weight and food intake were monitored weekly. Animal rooms were maintained at 20–22 °C with 30–70% relative humidity.

### Irradiation

At 5–6 months of age, mice were sub-lethally irradiated (NDT 320 or X-RAD225, 225 kV) with a total of 12 Gy of X-ray irradiation (3 times 4 Gy, with 2 days recovery between doses). Two days prior to irradiation (IR), and for 14 days post-IR, mice received 1% Baytril solution (Broad-spectrum antibiotic) in drinking water. At 1 month after IR, mice were sacrificed at the end of the study by cervical dislocation and tissues harvested, and stored in 4% paraformaldehyde for 24 h for paraffin embedding, or frozen in liquid nitrogen. Blood samples were isolated by cardiac puncture.

### Histological study

After sacrifice of mice, livers were excised and immediately stored in 4% paraformaldehyde at room temperature for 24 h for paraffin embedding after a brief rinse with PBS. The specimens were cut into 5-μm sections and stained with hematoxylin and eosin. The images were evaluated by a pathologist to find possible damages.

### Immunofluorescent staining of paraffin-embedded sections

Paraffin sections were attached to superfrost plus slides (Menzel-Glaser, Braunschweig, Germany) at 60 °C for 1 h. Deparaffinization was performed by pure xylol washes 3 times for 10′ each. Slides were rehydrated by ethanol solutions immersion (from 100 to 70%) for 5′ each and rinsed with deionized water. For the heat antigen retrieval, slides were immersed in sodium citrate 10 mM (unmasking solution) and microwaved at 800 W for 15′, then samples were kept at room temperature until cool down. Slides were rinsed with PBS 1 × 3 times, and then blocking solution (2% BSA, 0.05% Triton X-100 in PBS 1×) was applied for 1 h. The samples were surrounded with a hydrophobic barrier using a barrier pen, and the primary antibody (p16) was applied at 1:100 concentration and diluted in a blocking solution overnight. The next day, slides were rinsed 3 times with PBS 1×, and the secondary antibody was applied at 1:400 concentration diluted in blocking solution for 2 h. Again, slides were rinsed 3 times with PBS 1×, and DAPI staining (1 µg/ml) was applied for 10′. Finally, samples were mounted with coverslips using Vectashield Mounting Medium (Vector Laboratories, Burlingame, CA, USA, H1000). Presence of positive p16 cells were quantified.

### Cell culture

Primary human fibroblasts (Thermo fisher; C0135C) were cultured in high glucose DMEM (Dulbecco’s modified media) (Gibco, Invitrogen, Eugene, OR, USA) supplemented with 10% fetal bovine serum (FBS) (Gibco, Invitrogen, Eugene, OR, USA) and antibiotics (Sigma Chemical Co., St. Louis, MO, USA). Cells were incubated at 37 °C in a 5% CO2 atmosphere. Senescent cells were generated by X-ray irradiation. 6300 cells/cm2 were seeded 24 h prior to 20 Gy irradiation and were used for experiments 7 days later.

### Conditioned medium

Irradiated (20 Gy) or non-irradiated cells (2 × 10^6^) were seeded in a 10 cm dish and incubated for 7 days in DMEM with 0.5% FBS. After incubation, the conditioned medium (CM) was collected, centrifuged at 5000 g and filtered through a 0:2 µm pore filter. CM was mixed with DMEM 40% FBS in a proportion of 3–1 to generate CM containing 10% FBS.

### Immunofluorescence assay

Fibroblasts were grown on 1 mm width glass coverslips for 72 h in high glucose DMEM medium containing 10% FBS and 1% antibiotics. They were washed twice with PBS, fixed in 3.8% paraformaldehyde for 15′ at room temperature, permeabilized with 0.1% Triton X-100 in PBS for 10′ and incubated in blocking buffer (BSA 1%, 0.05% Triton X-100 in PBS) for 30′. In the meantime, the primary antibody was diluted 1:100 in antibody buffer (0.5% BSA, 0.05% Triton X-100 in PBS). Fibroblasts were incubated overnight at 4 °C with the primary antibody and subsequently washed twice with PBS. The secondary antibody was similarly diluted 1:400 in antibody buffer, but their incubation time on cells was reduced to 2 h at room temperature. Coverslips were then washed twice with PBS, incubated for 5′ with PBS containing DAPI 1 µg/ml and washed again with PBS. Next, they were mounted on microscope slides using Vectashield Mounting Medium (Vector Laboratories, Burlingame, CA, USA, H1000).

### SA-β-galactosidase assay and immunofluorescence

5 × 10^4^ IMR90 cells per well were seeded in 6-well plates. Four days later, cells were fixed with 0.5% glutaraldehyde (Sigma) in PBS for 10 min. Fixed cells were washed three times with PBS 1 mM MgCl_2_ pH 5.7, before adding to each well 2 ml of pre-warmed X-Gal staining solution (2 mM MgCl_2_, 5 mM K_4_Fe(CN)_6_—3H_2_O, 5 mM K_3_Fe(CN)_6_, 1 mg/ml X-Gal solution ready to use (R0941, ThermoFisher) in PBS). Plates were incubated for 2–24 h at 37 °C, washed and imaged. SA-β-Gal activity positive and negative cells were quantified using FIJI/ImageJ.

Healthy control, from BioChain (T1234149), and fresh patient liver tissues were fixated with Aceton for 10 min (4℃). Slides were rinsed 3 times with PBS 1× and blocking solution (2% BSA, 0.05% triton X-100 in PBS 1×) was applied for 1 h. Samples were surrounded by a hydrophobic barrier using a barrier pen. Primary antibody (Anti-beta Galactosidase (ab9361) and Anti-Ki67 (ab15580) antibodies, Abcam) were applied in blocking solution overnight (4℃). The next day, slides were rinsed 3 times with PBS 1×. Secondary antibody was applied in blocking solution for 2 h (RT). Again, slides were rinsed 3 times with PBS 1× and DAPI staining solution (1 µg/ml) was applied for 10 min. Samples were mounted on coverslips in using Prolong® Gold Anti Fade Reagent.

### ELISA (enzyme-linked immunosorbent assay)

IL-6, IL-8, IL-1β and IL-18 levels were assayed in supernatant by duplicate using commercial ELISA kits (Thermo Fisher Scientific, MA, USA).

### Cytokine array

Blood serum was collected from wild‐type and *Nlrp1*, *Nlrp3* and *Gsdmd*^−/−^ mice. In an in vitro model of senescence induced by X‐ray irradiation (10 Gy) (with or without irradiation), cells were cultured in serum‐free media for 24 h and media were collected for analysis. Media and blood serum were analyzed for expression of several mouse cytokine and chemokines (MD44) or human cytokine and chemokines (HD48), respectively, using a Multiplexing LASER Bead Assay (Eve Technologies).

### siRNA transfection

Cells were seeded on 6-well plates until 75% confluence in 2 ml DMEM high glucose medium (Cat. 10566016) supplemented with 10% FBS and 1% antibiotics. Transfection was performed according to the lipofectamine RNAiMAX reagent (Cat. 13778–075) protocol. Briefly, the siRNA-lipid complex was prepared in DMEM medium with 3% lipofectamine and 30 pmol of the correspondent siRNA, and incubated for 5 min at RT to form the silencing complex. Then, 250 µl of the siRNA-lipid complex were added to each well. After 72 h, cells were treated and analyzed for the different conditions. Every reagent, including DMEM medium, was purchased from ThermoFisher (Waltham, MA, USA).

### DNA treatment

For DNA extraction, cells were seeded on T75 flasks until 80% confluence, then cells were irradiated (20 Gy irradiation and were used for experiments 7 days later) using X-RAD225, 225 kV. After 1 week, irradiated and control cells were scrapped off and centrifuged at 1.000 g for 5′. DNA extraction was performed using 500 μl lysis buffer containing 100 mM Tris HCl, 100 mM EDTA, 100 mM NaCl, SDS 1%, pH 7.5. Samples were incubated at 65 °C for 30′, and 500 μl Phenol/Chloroform/Isoamyl Alcohol 25:24:1 was added. Samples were mixed by simple inversion and centrifuged at 5.000 g for 5′. 300 μl DNA-containing top aqueous phase was retrieved. For DNA precipitation, samples were mixed with 200 μl 5 M potassium acetate and 400 μl isopropanol. After 12.000 g for 15′ centrifugation, the pellet was washed with 70% ethanol twice and dried for 1 h. DNA pellet was resuspended in TE buffer (10 mM Tris HCl, 1 mM EDTA, pH 7.5) and quantified using a NanoDrop™ One/One from Thermo Fisher (Waltham, MA, USA).

Non-irradiated and irradiated DNA-treated cells were seeded on 6-well plates until 90% confluence in 2 ml DMEM high glucose medium supplemented with 10% FBS and 1% antibiotics. Then, 1 µg/ml of control/irradiated DNA was added for 8 h/24 h. Finally, cells were scrapped off and pelleted for further analysis.

Poly(dA-dT) DNA was transfected using Lipofectamine 2000 at 1 µg/ml concentration for 24 h. Cells were scrapped off and pelleted for further analysis.

### Immunoblotting

Western blotting was performed using standard methods. After protein transfer, the membrane was incubated with various primary antibodies diluted 1:1000; the corresponding secondary antibodies were coupled to horseradish peroxidase at a 1:10,000 dilution. Specific protein complexes were identified using the Immun Star HRP substrate kit (Biorad Laboratories Inc., Hercules, CA, USA).

### Bioinformatics analysis

We used ARCHS4 [12] to download the samples of the two datasets analyzed referring to mutations in NLRP1 and a model of oncogene-induced senescence in keratinocytes, GSE85791 & GSE180361, respectively. ARCHS4 provides RNAseq data that are uniformly processed using the Kallisto aligner [13]. Filtering and normalization were performed using the TMM method in EdgeR (3.40.1). Differentially expressed genes were obtained using the VOOM function from the Limma package (3.54.0). An FDR < 0.05 was used as a cut-off. Finally, differentially expressed genes from both datasets were individually selected for Gene Set Enrichment Analysis (GSEA) using GSEA from the GSEABase package (1.6.0), selecting hs_gsea_c2 as the geneset.

### Statistics

All data are expressed as means ± SEM. After evaluation of normality using Shapiro–Wilk test, statistical differences among the different groups were measured using either an unpaired Student *t* test or 1-way analysis of variance (ANOVA) when appropriate with Tukeys post-hoc test. A *P* value of ≤0.05 was considered statistically significant. Statistical analyses were performed using Prism software version 5.0a (GraphPad, San Diego, CA). Asterisks in the figures represent the following: * *P* ≤ 0.05; ** *P* ≤ 0.01; and *** *P* ≤ 0.001.

## Results

### NLRP1 expression is stimulated during senescence

**Fig. 1:**
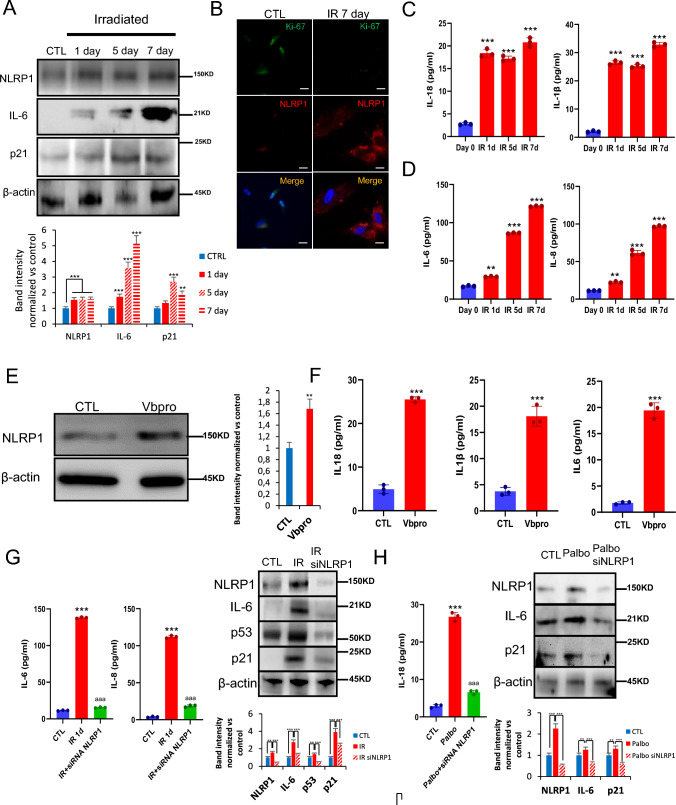
NLRP1 expression is associated with senescence. **A** Human fibroblasts were exposed to 20 Gy ionizing irradiation (IR). On day 1, 5 and 7, NLRP1 and senescence protein expression were analyzed by immunoblotting. **B** Representative images of Ki67 and NLRP1 immunofluorescence. Scale bar = 50 μm. **C**,**D** IL-1β, IL18, IL-6 and IL-8 were quantified by ELISA. All data are presented as means ± SEM, *n* = 4 independent experiments; ** *P* < 0.005, *** *P* < 0.001 differences between time points after irradiation and day 0. **E**,**F** Human fibroblasts were treated with Valboropro (Vbpro) to induce NLRP1 expression. After 24 h, NLRP1 and IL-6 protein expression were analyzed by immunoblotting and cytokines were analyzed by ELISA. Human fibroblasts were irradiated (**G**) or stimulated with palbociclib (Palbo) (**H**) to induce two different senescence models. Then, cells were transfected with a non-targeting control siRNA (Control) or with siRNAs against NLRP1 (siNLRP1). Expression of NLRP1 and senescence-associated proteins p16, p21, p53 and IL-6 were assessed by immunoblotting and IL6, IL-8 or IL-18 were quantified by ELISA. All data are presented as means ± SEM, *n* = 4 independent experiments; ** *P* < 0.005, *** *P* < 0.001 irradiated vs. control. ^aaa^ *P* < 0.001, IR + siRNA vs. IR cells (color figure online)

The ability of irradiation to cause cellular senescence provides an opportunity to study the role that NLRP1 and NLRP3 play in this process. To validate the presence of senescent cells associated to inflammasomes we used the established senescence detection multimarker algorithm using different biomarkers and procediments [14]. Irradiation of human fibroblasts induced sustained expression of NLRP1, NLRP3, and senescence-associated proteins, p21 and IL-6, but temporal AIM2 expression (Fig. 1A; Fig. S1). Analysis of the proliferation marker Ki67 confirmed that fibroblasts with reduced Ki-67 also had increased NLRP1 expression after 7 days of irradiation (Fig. 1B; Fig. S2). Irradiation also stimulated the expression of IL-1β, IL-6, IL-8, and IL-18 as detected by ELISA (Fig. 1C, D). Immunofluorescence experiments also showed that NLRP1 positive cells had high colocalization with the SASP marker IL-6 (Fig. S3A and B). To reinforce the role that NLRP1 plays in SASP expression, we treated skin fibroblasts with ValboroPro (Vbpro), a potent inhibitor of dipeptidyl peptidase 9 (DPP9), which interferes with NLRP1 expression [15, 16]. Vbpro stimulated the expression of NLRP1, IL-1β, IL-18 and IL-6 (Fig. 1E, F). To confirm that SASP production is NLRP1-dependent, we determined the effects of siRNA-mediated NLRP1 knock-down in fibroblasts. NLRP1 knock-down inhibited the expression of p21, p53, IL6, IL-8, cleaved GSDMD and IL1β (Fig. 1G; Fig. S4A and B) and downregulated IL-6 and IL-8 medium release levels. NLRP3 knock-down also inhibited the expression of p53, IL-6 and cleaved GSDMD but to a lesser extent compared to NLRP1 deficiency (Fig. S4A and B). These results suggest the NLRP1 inflammasome is the major regulatorof SASP in response to irradiation.

As a typical response of the cell during irradiation Caspase 3 protein levels were increased together with the pyroptotic protein Gasdermin D (GSDMD) which was associated to a moderate increase of lactate dehydrogenase (LDH) release showing a pyroptotic cell death. GSDMD activation and pyroptosis are directly associated to NLRP1 and inflammasome activation [4]. Interestingly, NLRP1 knock-down reduced both Caspase 3 and GSDMD protein expression ad LDH release (Fig. S5A and B). These results suggest the NLRP1 inflammasome regulates SASP and senescent cell death by pyroptosis and not NLRP3 inflammasome.

To determine whether the activation of NLRP1 is a common feature of different senescence inducers, we exposed human fibroblasts to palbociclib (PD-0332991), a CDK4 inhibitor, known to mimic the effect of p16^Ink4a^ [17–19]. Palbociclib induced the expression of IL-18, NLRP1, IL-6, and p21, responses that were reduced in cells in which NLRP1 levels were down regulated by siNLRP1 (Fig. 1H). Thus, the expression of NLRP1 is stimulated by two different senescence activators and drives the SASP in both cases.

### NLRP1 inflammasome is necessary to induce paracrine bystander senescence

**Fig. 2:**
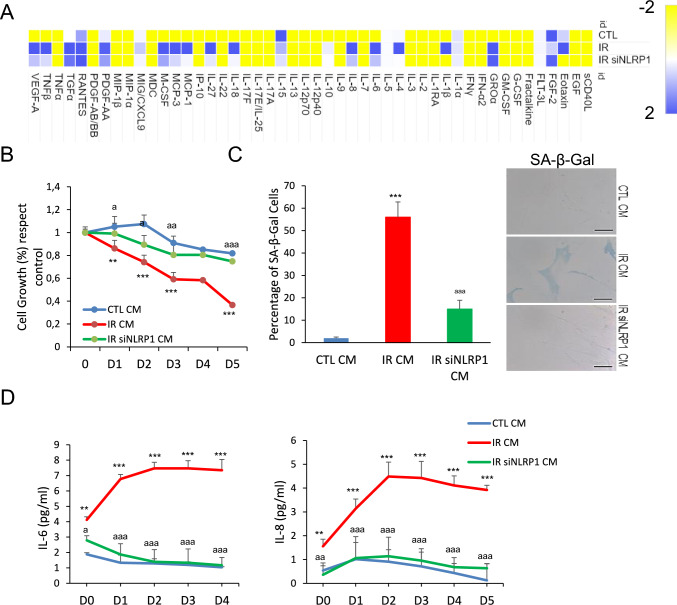
NLRP1 is necessary for paracrine senescence. **A** Heat map depicting expression of 48 human cytokines in the medium at 7 days following culture from IR and IR + siRNA NLRP1. SASP was measured from the supernatant of the cells. **B** Effect of conditioned medium (CM) in human fibroblast growth. CM was collected from control, IR or IR + siNLRP1 cells. Percentage of cell growth was determined over 5 days. Image show cell populations stained with DAPI. **C** The induction of SA-β-Gal activity by CM was determined by microscopy. **D** IL-6 and IL-8 cytokine release to the medium after culture with CM from control, IR or IR + siNLRP1 cells. All data are presented as means ± SEM, *n* = 4 independent experiments; ** *P* < 0.005, *** *P* < 0.001 irradiated vs. control. ^a^ *P* < 0.05, ^aa^ *P* < 0.005, ^aaa^ *P* < 0.001, IR + siRNA vs. IR cells (color figure online)

The NLRP1 inflammasome has been described as a sensor of various types of perturbations, modulating inflammation by mediating the secretion of pro-inflammatory cytokines, IL-18 and IL-1β [15, 20]. We hypothesized that the NLRP1 inflammasome promotes senescence by regulating the SASP. To test this hypothesis, we exposed human skin fibroblasts to conditioned medium (CM) of human skin fibroblasts exposed to irradiation (supernatants from fibroblasts cultured for 7 days-post irradiation). Cytokine array analysis of a panel of SASP components showed that CM induced SASP expression in skin fibroblasts, a response that was attenuated in cells transfected with siNLRP1 (Fig. 2A). CM from irradiated cells also impaired cell growth and promoted senescence as indicated by increased SA-Gal activity, IL-6 and IL-8 secretion, outcomes that were also decreased in siNLRP1-exposed cells (Fig. 2B–D). These data strongly suggest that irradiation of human fibroblasts induced SASP factors through the NLRP1 signalling pathway.

### NLRP1 deletion reduces senescence in vivo

**Fig. 3:**
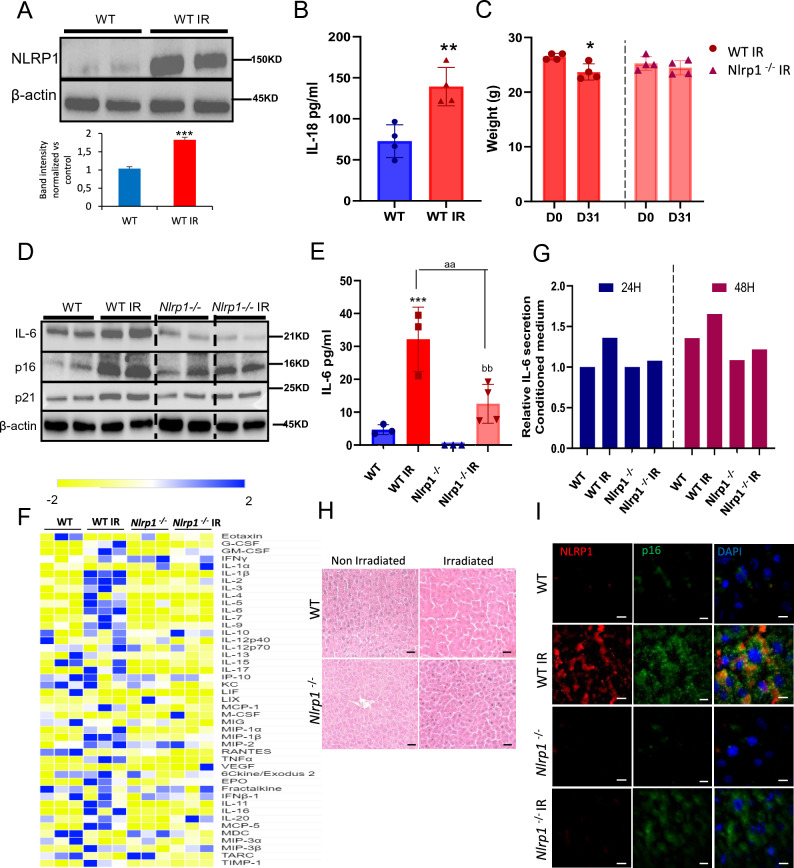
NLRP1 contributes to cellular senescence in vivo. **A** *Nlrp1* protein expression in liver from WT mice at 1 month after IR. **B** Serum levels of IL-18 after IR. **C** Effect of IR on the bodyweight of WT and *Nlrp1* knockout (KO) mice. **D** Protein expression in liver from IR and non-IR WT and *Nlrp1* KO mice of senescent markers (IL-6, p16 and p21). Densitometry in Supplementary Fig. 6. **E** Serum levels of IL-6 in serum from IR and non-IR WT and *Nlrp1* KO mice. **F** Heat map depicting expression of 44 mouse cytokines in serum at 5 weeks after IR of WT and *Nlrp1* KO mice. *n* = 6 mice per group. **G** IL-6 releases from healthy fibroblasts was assessed after 24 and 48 h of incubation with media containing serum from IR and non-IR WT and *Nlrp1* KO mice. **H** Representative liver section stainings of hematoxylin and eosin (H&E). **I** Representative liver section immunostainings of NLRP1 and p16. All data are presented as means ± SEM, *n* = 6–8 mice per group; * *P* < 0.05, ** *P* < 0.005, *** *P* < 0.001 irradiated vs. control. ^aa^ *P* < 0.005 IR WT vs. IR KO mice; ^bb^ *P* < 0.005 IR KO vs. IR KO mice (color figure online)

To determine whether NLRP1 regulation of senescence occurs in vivo, we exposed wild-type (WT) mice and mice lacking all three alleles of NLRP1 (*Nlrp1a*^−/−^*Nlrp1b*^−/−^*Nlrp1c*^−/−^, referred to as *Nlrp1*^−/−^) to total body irradiation and analyzed senescence- and SASP expression. First, we corronorate if NLRP1 expression was increased in livers from irradiated WT mice alongside serum IL-18 levels 1 month after irradiation similar to our in vitro experiments (Fig. 3A, B). WT mice showed a moderate body weight loss after irradiation, an outcome that was not observed in *Nlrp1*^−/−^ mice (Fig. 3C). WT mice also exhibited increased levels of IL-6, p16, p21, and several inflammatory mediators in the liver, outcomes that were decreased in *Nlrp1*^−/−^ mice (Fig. 3D–F; Fig. S6). To confirm the modulatory effects of NLRP1 on SASP, human fibroblasts were exposed to serum from irradiated mice. WT serum induced higher IL-6 levels compared to *Nlrp1*^−/−^ serum (Fig. 3G). Irradiation caused liver damage with sinusoidal dilatation, oedema and mononuclear cell recruitment (Fig. 3H) and increased nuclear and cytosolic p16^Ink4a^ expression in NLRP1-expressing cells (Fig. 3I), responses that were prevented by NLRP1 deletion.

We also determined the role of NLRP3 on SASP secretion in the irradiation model. Unlike *Nlrp1*^−/−^ mice, *Nlrp3*^−/−^ mice showed significant weight loss after irradiation similar to WT mice (Fig. S7A). Irradiation also induced senescence and SASP markers p16, p21 and IL-6 to a similar extent in WT and *Nlrp3*^−/−^ mice (Fig. S7B–D). Together, these data indicate that the NLRP1 inflammasome, but not NLRP3 inflammasome controls irradiation-induced senescence and SASP in vivo.

### NLRP1 controls senescence in human tissues

**Fig. 4:**
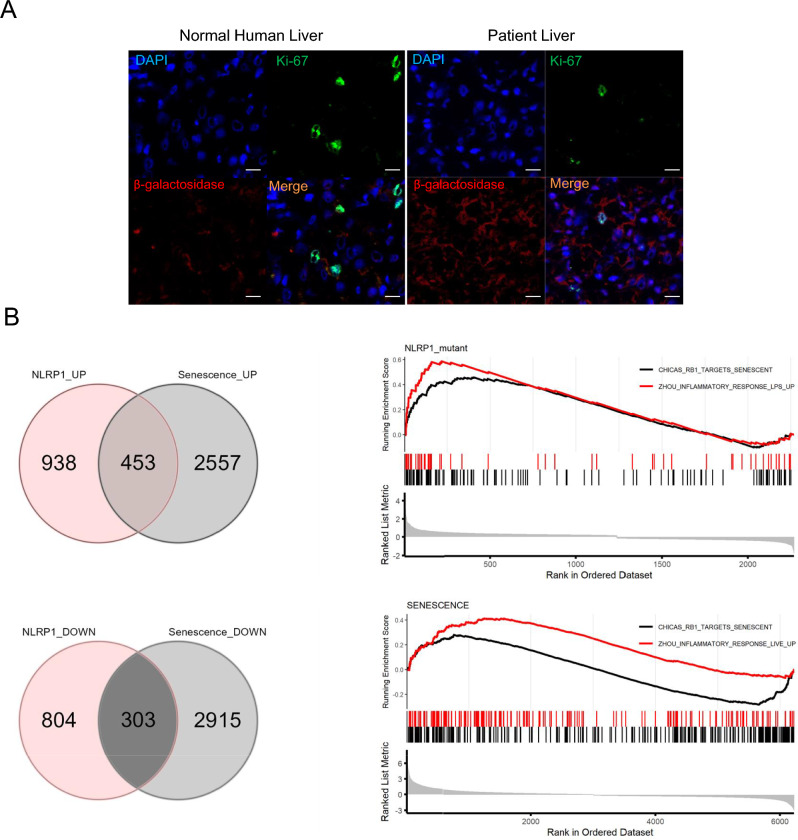
NLRP1 is associated with senescence in humans. **A** β-Gal and Ki-67 immunostaining in liver section from a patient with a NLRP1 gain of function mutation. **B** Venn Diagrams showing genes that are changed in both selected datasets (left), and genes that are upregulated (middle) or downregulated (right). GSEA analysis showing two gene datasets enrichered for inflammatory response and targets of senescences genes is shown below (color figure online)

To determine the relevance of NLRP1-mediated senescence in humans, we first examined p16^Ink4a^ expression in liver of a patient with NLRP1-dependent inflammasome hyperactivation caused by de novo c.3641C>T (p.P1214L) mutation in *NLRP1* [21]. The patient showed clinical features associated with hyperkeratosis, liver cirrhosis and increased serum IL-18 but not of IL-1β. Immortalized hepatocytes from the patient released higher IL18 and lower levels of IL1β, respectively with respect to healthy controls [21]. Accordingly, we found increased β-galactosidase expression and reduced Ki-67 cells in liver sections of this patient compared to a control liver samples (Fig. 4A), findings that are consistent with the previously reported association of senescence with liver steatosis and cirrhosis [22–25].

Further, we compared the transcriptomes of keratinocytes from patients with germline mutations in *NLRP1* [26] versus a model of oncogene-induced senescence of these cells. We found that 756 transcripts were similar between both datasets, with 453 and 303 transcripts being up- and down-regulated, respectively. Furthermore, GSEA analysis revealed significant up-regulation of two sets of genes involved in the inflammatory responses and senescence (Fig. 4B). These results suggest that activation of the NLRP1 inflammasome triggers a transcriptomic reprogramming that is similar to the one occuring during senescence in humans.

### NLRP1 is activated by damaged DNA

**Fig. 5:**
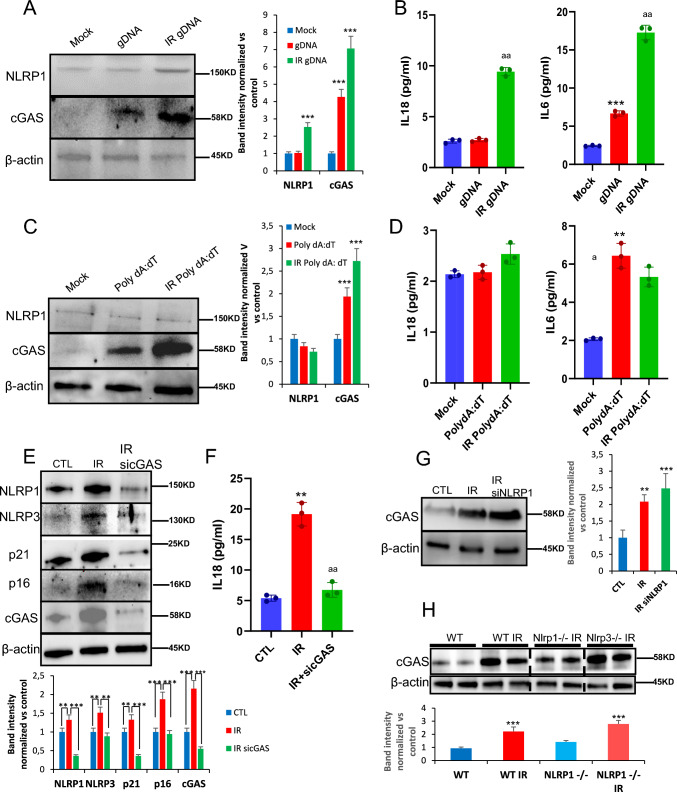
NLRP1 senses DNA damage dependent of cGAS activation. **A** Protein expression levels of NLRP1 and cGAS after 24 h exposition to gDNA from non-irradiated and irradiated cells. **B** IL-6 and IL-18 release to the medium after the same experimental condition. Levels were determined by ELISA assay. **C**,**D** Protein expression levels of NLRP1 and cGAS and IL-6 and IL-18 release after 24 h exposition to a non-irradiated or irradiated synthetic double-stranded DNA sequence, poly(dA-dT), Cytokine levels were determined by ELISA assay. Cytokine levels were determined by ELISA assay. All data are presented as means ± SEM, *n* = 4 independent experiments; ** *P* < 0.005, *** *P* < 0.001 gDNA vs. control cells. ^a^ *P* < 0.05, ^aa^ *P* < 0.005, ^aaa^ *P* < 0.001, IR gDNA vs. control cells. **E** Human fibroblasts were irradiated to induces senescence. Then, cells were transfected with a non-targeting control siRNA (Control) or with siRNAs against cGAS (sicGAS). Expression of NLRP1, NLRP3, IL-1β, cGAS and senescent protein p16, p21 was assessed by immunoblotting. **F** IL-18 release of non-irradiated, irradiated and irradiated and transfected with siRNAs against cGAS. Cytokine levels were determined by ELISA assay. All data are presented as means ± SEM, *n* = 4 independent experiments; ** *P* < 0.005, IR vs. control cells. ^aa^ *P* < 0.005, IR vs. sicGAS (color figure online)

DNA damage is commonly associated with senescence upon exposure to stressors such as ROS, radiotherapy, and chemotherapy [18], which is detected by various sensors, including cGAS [21]. NLRP1 is known for sensing double-stranded (ds) RNA, but not long dsDNA molecules [20, 21, 26]. Given the role of NLRP1 as mediator of senescence and SASP as outlined above, we hypothesized that the NLRP1 inflammasome might be activated by damaged DNA. Therefore, we analyzed NLRP1 inflammasome-dependent responses upon exposure to human genomic DNA from irradiated cells (IR gDNA). For comparison, we also used poly(deoxyadenylic-deoxythymidylic) acid (poly dA:dT) to activate the AIM2 inflammasome [27]. IR gDNA addition increased NLRP1 and cGAS protein expression compared to non-IR gDNA (Fig. 5A), a response that correlated with IL-6 and IL-18 release (Fig. 5B). Moreover, poly(dA:dT) oligonucleotide induced a significant overexpression of cGAS with IL-6 release, but failed to induce NLRP1 expression or IL-18 release (Fig. 5C, D). These results suggest that NLRP1 expression is increased in response to DNA damage.

To investigate whether NLRP1 expression depends on cGAS modulation, we knocked down cGAS with siRNAs in fibroblasts, which were then subjected to irradiation. Down regulation of cGAS resulted in decreased expression of not only p21 and p16 but also NLRP1, NLRP3, and IL-18 (Fig. 5E, F), suggesting that NLRP1 might modulate senescence downstream of cGAS with more potential effect that NLRP3. This view was supported by the fact that similar to NLRP1, cGAS expression was stimulated by irradiation (Fig. 5G, H). We noticed that lack of NLRP1 and NLRP3 led to increased cGAS expression (Fig. 5G), suggesting a compensatory effect on cGAS in the absence of NLRP1, findings that are consistent with the previously reported modulation of senescence and NLRP3 by cGAS pathway [18, 21, 28]. These observations strengthen our hypothesis that NLRP1 expression is positively regulated by cGAS.

### NLRP1 controls SASP release through GSDMD pores

**Fig. 6:**
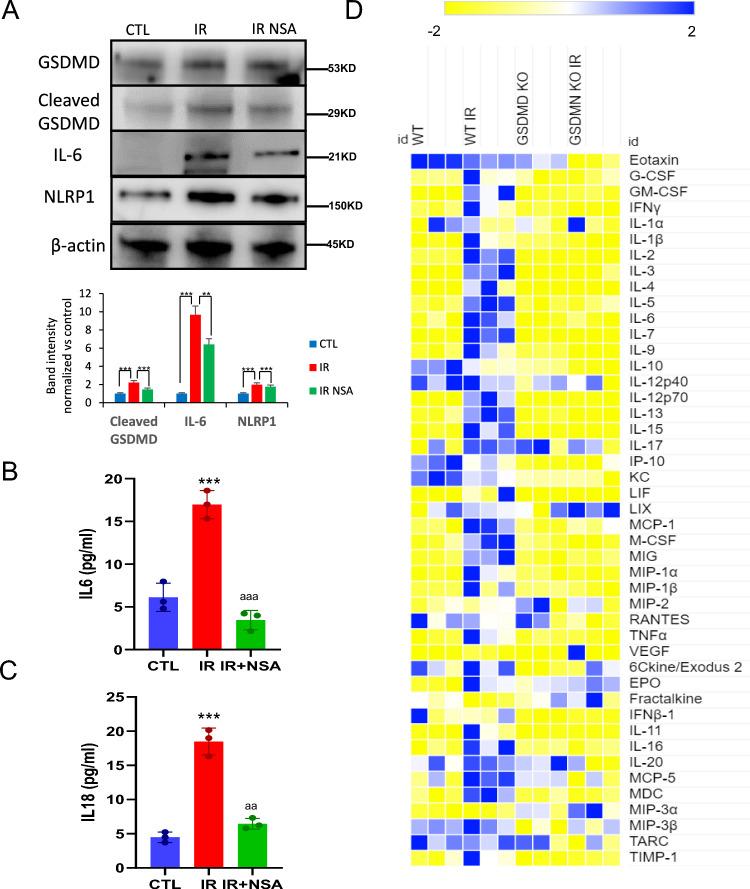
Gasdermin D mediates SASP release. **A** Protein expression levels of GSDMD, its cleaved form, IL-6 and NLRP1 in fibroblasts which were irradiated (IR) and treated with necrosulfonamyde (NSA) for 5 days after IR. **B**,**C** IL-6 and IL-18 release determined by ELISA assay. Data are presented as means ± SEM, *n* = 5 independent experiments; *** *P* < 0.001 irradiated vs. control. ^aa^ *P* < 0.005 IR cells vs. IR NSA cells. **D** Heat map depicting expression of 44 mouse cytokines in serum at 5 weeks after IR of WT and *Gsdmd* KO mice *n* = 5 mice per group (color figure online)

GSDMD pores release not only IL-1β and IL-18 [18], but also IL-33, another SASP member [29, 30]. Irradiation induced GSDMD cleavage in human fibroblasts, a response that was reduced by the GSDMD inhibitor necrosulfonamide (NSA) [30]. Irradiation-increased GSDMD expression and cleavage, responses that correlated with increased IL-6 expression (Fig. 6A). NSA treatment reduced irradiation-induced GSDMD cleavage and cellular IL-6 abundance (Fig. 6A). NSA treatment completely blocked IL-6 and IL-18 secretion (Fig. 6B, C). Finally, we explored the role of GSDMD in SASP release in vivo by exposing *Gsdmd* KO (*Gsdmd*^−/−^) mice to total body irradiation (3 times 4 Gy, with 2 days recovery between doses) and assessing a panel of SASP components in serum by cytokine array. Irradiation increased serum levels of various inflammatory factors, including SASP in WT mice, responses that were largely abrogated in *Gsdmd*^−/−^ mice (Fig. 6D). These results suggest that the absence of GSDMD pores blocks the release of SASP factors in response to irradiation.

## Discussion

We demonstrated that NLRP1 inflammasome acted downstream of cGAS to regulate senescence through GSDMD-dependent SASP release in various in vitro and in vivo models. cGAS recognizes diverse DNA species, including self-DNA [31, 32], and its interactions with NLRP1 during senescence has not been reported. Our data suggest that damaged DNA activates cGAS-mediated SASP and senescence upon irradiation. This response is mediated by the NLRP1 inflammasome GSDMD pathway. Our observations are consistent with recent works showing that the human NLRP1 inflammasome mediates skin damage caused by ultraviolet (UVB), which also induces senescence [33–35]. Our findings were also consistent with the association of NLRP1 overexpression or gain-of-function mutations with the prevalence of skin diseases such as psoriasis, vitiligo, atopic dermatitis or hyperkeratosis [36] where senescence had been widely described [37, 38].

Our study shows increased levels of the senescence marker, β-Galactosidase and reduced postitive Ki-67 cells, in the liver of a patient with a NLRP1 gain-of-function mutation. Furthermore, the transcriptome of NLRP1 gain-of-function mutations in human cells shows a significant inflammatory phenotype. This phenotype is associated with upregulation of stress-responsive secreted factors and known pro-inflammatory cytokines associated with SASP [39]. The DPP8/DPP9 inhibitor Talablostat, also named PT-100 (Vbpro), has been shown to induce human and rodent NLRP1 inflammasomes activation, and stimulate the expression of SASP [40–43]. Similarly, Maver et al. [39] described homozygous missense variant in NLRP1 (Gly587Ser) in a family with multiple sclerosis with high IL-1β levels associated with several senescence and aging-associated genes such as NFKB, JNK and p38 pathways [39]. Furthermore, Zhong et al. [25] reported that germline NLRP1 mutations cause skin inflammation and cancer susceptibility [25].

The NLRP1 inflammasome, like the NLRP3 inflammasome, has been associated with different age-related diseases [7, 8]. For example, increased protein levels of NLRP1 and caspase-1 is shown in the prefrontal cortex of mice during aging [44]. Further, NLRP1 has also been proposed to contribute to the effect of age on chronic stress-induced depressive-like behaviour in mice [45]. Furthermore, a recent study shows that the NLRP1 inflammasome is involved in age-related neuronal damage in which senescence markers was increased [46]. NLRP1-siRNA treatment reduces neuronal senescence and damage associated with decreased β-galactosidase and apoptosis [46].

In conclusion, our findings on NLRP1 provide new insights into the mechanism of SASP production during senescence. Although several NLRP3 inhibitors have been developed, specific NLRP1 inhibitors are not currently available. Our study provides a rationale for targeting NLRP1 to control cGAS-dependent inflammation and senescence responses that drive human diseases.

### Supplementary Information

Below is the link to the electronic supplementary material.

Supplementary file1 (PDF 439 KB)

## Data Availability

The datasets used and/or analyzed during the current study are available from the corresponding author on reasonable request.
