# Supplementary material for: NLRP1 inflammasome promotes senescence and senescence-associated secretory phenotype
Source: Inflamm Res. 2024 Jun 21;73(8):1253–66. doi: 10.1007/s00011-024-01892-7 (PMC11281979; doi:10.1007/s00011-024-01892-7)
Supplement: Supplementary file 1 — Supplementary file1 (PDF 439 KB) [file 11_2024_1892_MOESM1_ESM.pdf]

Supplementary Data

NLRP1 inflammasome promotes senescence and the senescence-associated secretory phenotype

Inés Muela-Zarzuela[1*], Juan Miguel Suarez-Rivero[1*], Andrea Gallardo-Orihuela[2*], Chun Wang[3], Kumi Izawa[4], Marta de Gregorio-Procopio[2], Isabelle Couillin[5], Bernhard Ryffel[5], Jiro Kitaura[4], Alberto Sanz[6], Thomas von Zglinicki[7], Gabriel Mbalaviele[3], Mario D. Cordero[7,8]

[1] Department of Molecular Biology and Biochemical Engineering, Universidad Pablo de Olavide, 41013 Seville, Spain.

[2] Instituto de Investigación e Innovación Biomédica de Cádiz, INiBICA, Hospital Universitario Puerta del Mar, Cádiz, Spain.

[3] Division of Bone and Mineral Diseases, Washington University School of Medicine, St. Louis, MO 63110, United States.

[4] Atopy (Allergy) Research Center, Juntendo University Graduate School of Medicine, Tokyo, Japan.

[5] Laboratory of Experimental and Molecular Immunology and Neurogenetics (INEM), UMR 7355 CNRS-University of Orleans, Orléans, France; IDM, University of Cape Town, South Africa

[6] School of Molecular Biosciences, College of Medical, Veterinary and Life Sciences, University of Glasgow, G12 8QQ, Glasgow, United Kingdom.

[7] Ageing Research Laboratories, Newcastle University, Biosciences Institute, Newcastle upon Tyne, United Kingdom

[8] CIBER de Enfermedades Respiratorias (CIBERES), Instituto de Salud Carlos III, 28220 Madrid, Spain.

* Co-first authors

Running Title: NLRP1-inflammasome modulates senescence

<u>Corresponding Author</u>:

Dr. Mario D. Cordero
Department of Molecular Biology and Biochemical Engineering, Universidad Pablo de Olavide, 41013 Seville, Spain. Email: mdcormor1@upo.es.

Keywords: NLRP1-inflammasome, senescence, SASP, aging

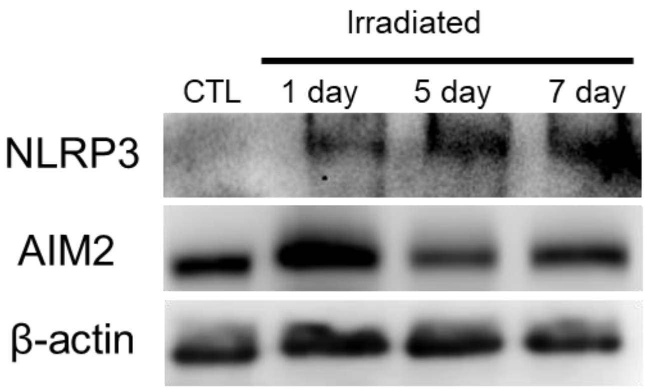

Supplementary Figure 1. NLRP3 expression is induced in a sustained manner during senescence induced by irradiation. At day 1, 5 and 7 NLRP3 and AIM2 expression was analysed by immunoblotting after irradiation. n = 4 independent experiments.

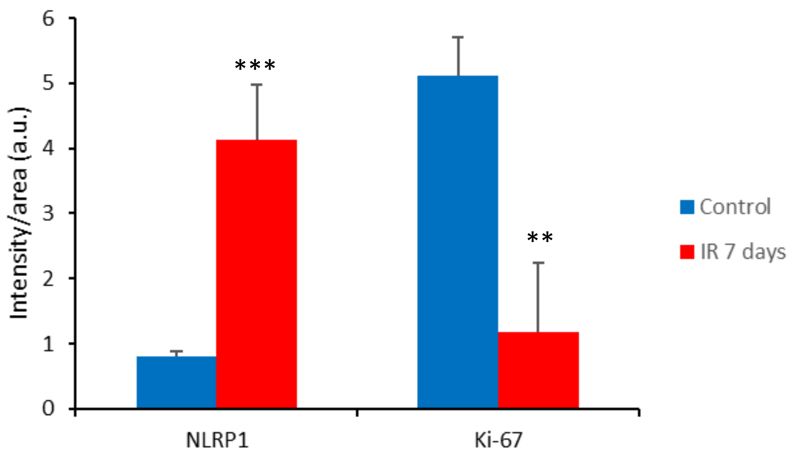

Supplementary Figure 2. Quantification of NLRP1 and Ki-67 fluorescence intensity levels from control and irradiated cells after 7 days. All data are presented as means ± SEM, n = 4 independent experiments; ***P < 0.001; **P < 0.01 irradiated *vs* control.

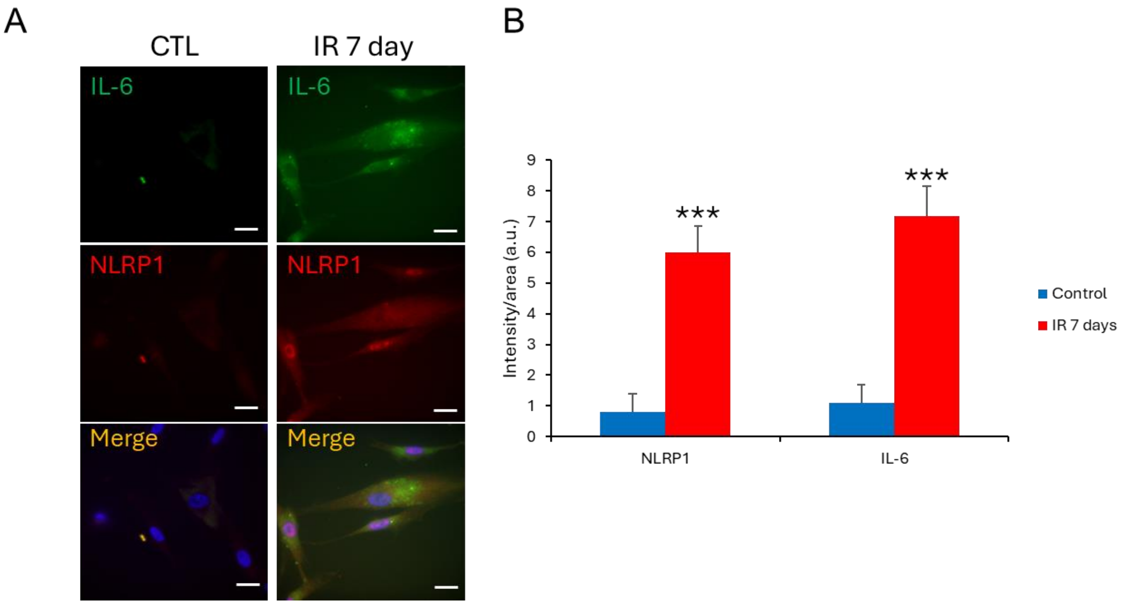

Supplementary Figure 3. Colocalization of NLRP1 and IL-6 SASP marker. A. Representative images of IL-6 and NLRP1 immunofluorescence. Scale bar = 50 µm. B. Intensity quantification of NLRP1 and IL-6 fluorescence levels from control and irradiated cells after 7 days. All data are presented as means ± SEM, n = 4 independent experiments; ***P < 0.001; irradiated *vs* control.

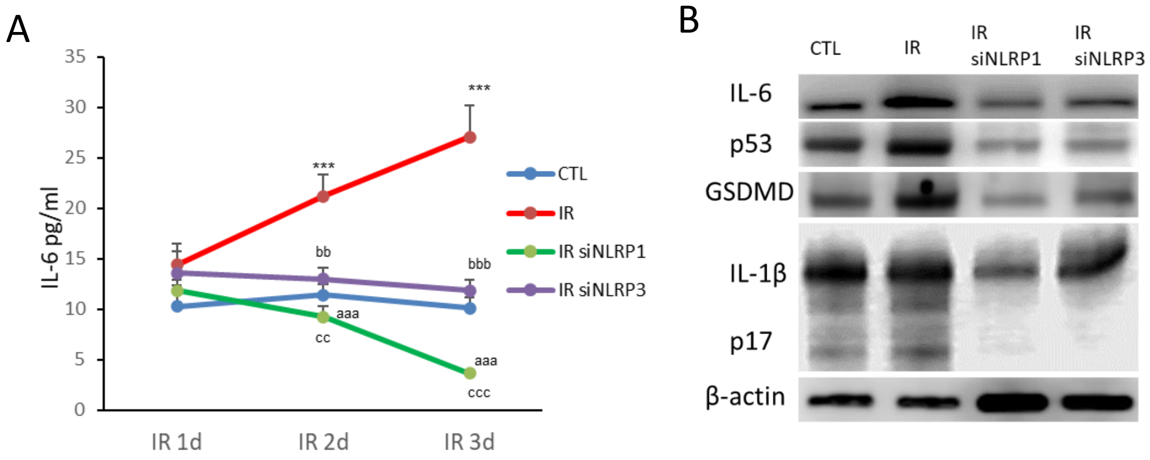

Supplementary Figure 4. NLRP1 induces a more significant senescence effect than NLRP3. (A) IL-6 release from cell IR, IR+siNLRP1 or IR+siNLRP3 cells and (B) protein expression. All data are presented as means ± SEM, n = 4 independent experiments; ***P < 0.001 irradiated *vs* control. ^{aaa}P < 0.001, IR+siRNA NLRP1 *vs* IR cells; ^{bb}P < 0.005, ^{bbb}P < 0.001, IR+siRNA NLRP3 *vs* IR cells; ^{cc}P < 0.005, ^{ccc}P < 0.001, IR+siRNA NLRP1 *vs* IR+siRNA NLRP3 cells.

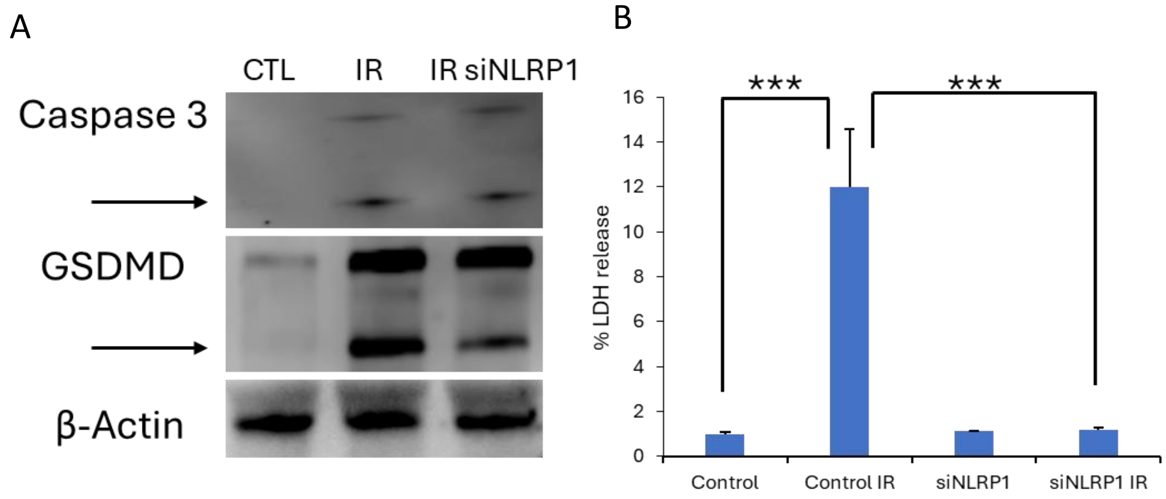

Supplementary Figure 5. Irradiation induces pyroptosis. A. Analysis of caspase-3 and Gasdermin D (GSDMD) after irradiation. B. Lactate dehydrogenase (LDH) release after irradation. All data are presented as means ± SEM, n = 3 independent experiments; ***P < 0.001.

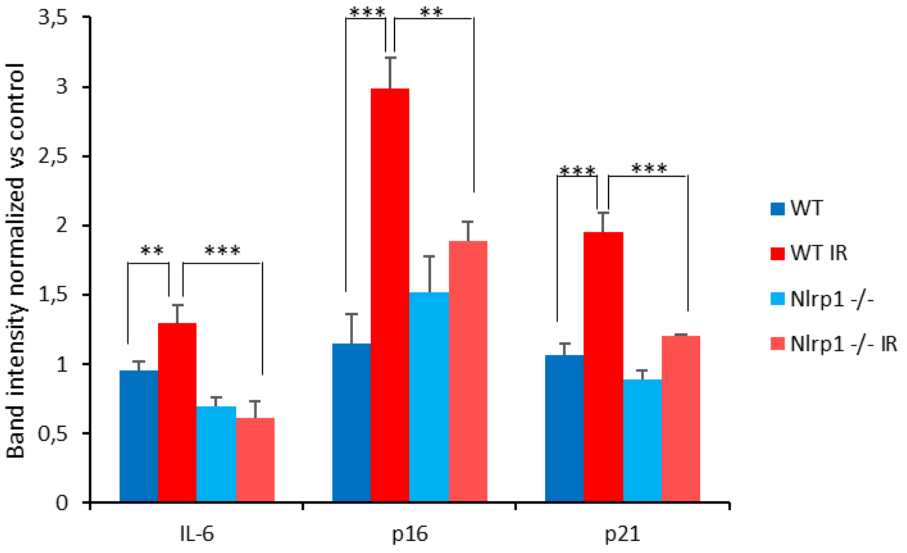

Supplementary Figure 6. Densitometric analysis of Figure 3D. Data are presented as means ± SEM, n = 3 independent experiments; ***P < 0.001; **P < 0.01.

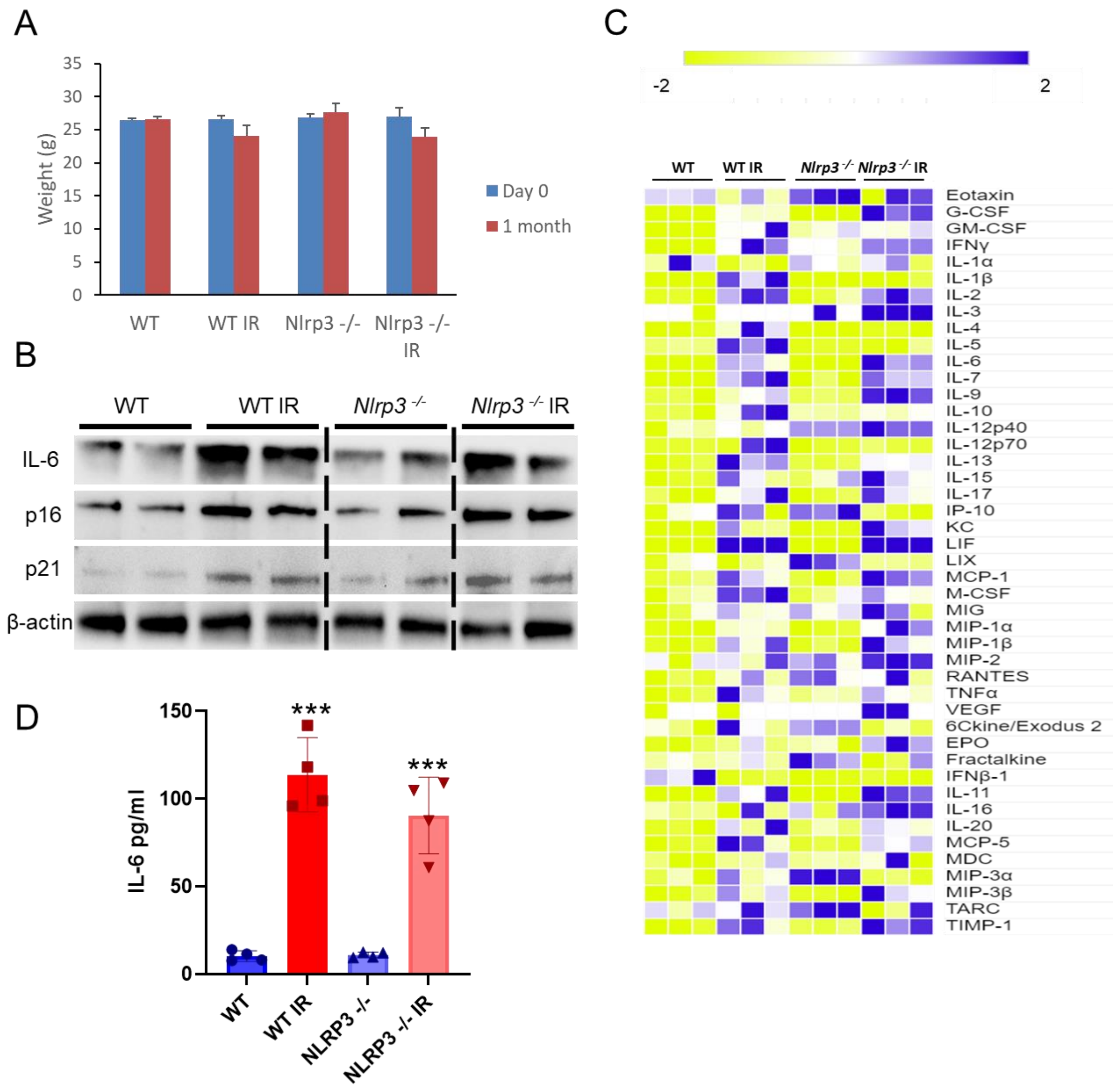

Supplementary Figure 7. NLRP3 deletion does not prevent senescence and SASP. (A) Effect of IR on the bodyweight of WT and NLRP3 knockout (KO) mice. (B) Protein expression in livers from IR and non IR-WT and NLRP3 KO mice of senescence protein markers (IL-6, p16 and p21). (C) Heat map depicting expression of 44 mouse cytokines in serum at 5 weeks following from IR of WT and NLRP3 KO mice. (D) IL-6 release from healthy fibroblasts was assessed after 24 and 48 hr of incubation with media containing serum from IR and non IR WT and NLRP3 KO mice. All data are presented as means ± SEM, n = 6-8 mice per group; ***P < 0.001 irradiated *vs* control.
